# High-frequency devices effect in vitro: promissing approach in the treatment of acne vulgaris?^☆^

**DOI:** 10.1016/j.abd.2021.09.015

**Published:** 2022-09-13

**Authors:** Leonie Frommherz, Markus Reinholz, Anne Gürtler, Pia-Charlotte Stadler, Till Kaemmerer, Lars French, Benjamin M. Clanner-Engelshofen

**Affiliations:** a Department of Dermatology and Allergy, Ludwig Maximilian University, Munich, Germany; b Dr Phillip Frost Department of Dermatology & Cutaneous Surgery, University of Miami, Miller School of Medicine, Miami, USA

**Keywords:** Acne vulgaris, Bacterial infection, High-frequency, Microbiome

## Abstract

**Background:**

Acne vulgaris is an inflammatory skin disorder leading to an impairment of quality of life and is therefore not only a cosmetic issue. Its pathogenesis is multifactorial – of particular importance is the colonization with the bacterium *Propionibacterium acnes*. A wide range of different treatment options exists including topical and systemic treatments depending on severity. High Frequency (HF) therapy, historically developed in the 19^th^ century, claims antimicrobial effects on acne skin, but solid data on its efficacy and mechanism of action is lacking.

**Objectives:**

The main objective of this study was to determine the efficacy of HF therapy on skin flora and *P. acnes* in vitro using a commercial device as well as to review studies on the mechanism of action.

**Methods:**

The plasma source was investigated regarding electrical settings, heat, and ozone development. Bacterial skin flora, fungal isolates, and *P. acnes* were exposed to HF in vitro and compared to unexposed controls by evaluating the number of colonies on agar plates. To further analyze bacterial species from normal skin flora, 16S-sequencing was performed. Statistical analyses were carried out using row analysis and unpaired *t*-test.

**Results:**

HF treatment led to a significant reduction of almost every bacterial and fungal species investigated in this study. Moreover, the number of colonies forming units was significantly decreased in *P. acnes* after HF treatment compared to controls in vitro.

**Study limitations:**

The experiments were performed in vitro only. To assess clinical effects further in vivo experiments are necessary.

**Conclusions:**

The results collected in this study, although in vitro, provide a mechanistic basis for HF as a complementary treatment option for patients with acne. It might also have a beneficial effect on patients with superficial infectious skin of the skin.

## Introduction

Acne vulgaris is frequently affecting approximately 80% of adolescents and is, therefore, one of the most common reasons for consultation with a dermatologist.^1^ The pathogenesis of this inflammatory skin disorder is multifactorial including increased sebum production, altered follicular keratinization, bacterial colonization with *Propionibacterium acnes,* and inflammation.^2^, ^3^, ^4^ It is divided into four grades from mild (including comedones and a few inflammatory papules) to severe (along with inflammatory nodules and scarring).^5^ Furthermore, acne is considered a stigmatizing disease leading to low self-esteem and social withdrawal, also reflected by a high Dermatology Life Quality Index (DLQI) resembling those of psoriasis vulgaris patients.^6^ Thus, the demand for effective treatments is particularly high. A multitude of treatment options exists ranging from over-the-counter medication to oral therapies including retinoids and antibiotics. Recently, high-frequency devices have gained increasing attention claiming to support lymph drainage, prevent both hair loss and the development of wrinkles, and ultimately, to improve acne lesions. Those high-frequency devices colloquially termed Violet Wand (VW), deliver Cold Atmospheric Pressure Plasma (CAPP). CAPP has multiple bioactive properties e.g., by the release of charged particles and reactive species (O_3_, NO, NO_2_).^7^ In the early last century, High Frequency (HF) therapy was already known as a versatile treatment option in patients with infectious diseases of the skin, eczema, and wounds. Additionally, even migraines, neuralgia, tuberculosis, and many more diseases were treated by this method.^8^ Probably due to the development of antibiotics and lacking data on efficacy, it became less important in the middle of the last century. Not only because of its antimicrobial effects as a “physical antiseptic” but also because of the global increase of multi-resistant bacterial strains, plasma medicine is currently an emerging field and ongoing studies are ubiquitous. Recently, it was shown that HF devices are more effective than common antiseptics in targeting wound pathogens *in vitro*,^7^, ^8^ arising the question of whether HF therapy may be also beneficial in acne-prone skin and how it affects common skin flora.

## Materials and methods

### Plasma source

**Fig. 1:**
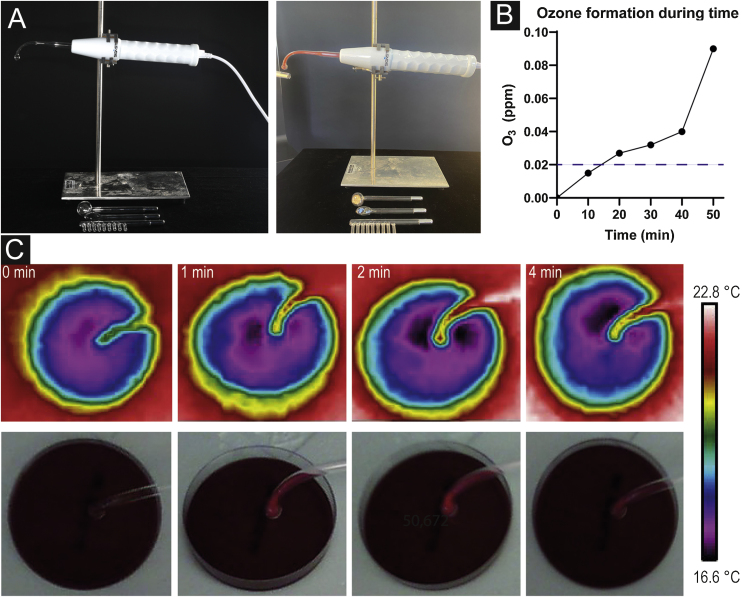
Plasma source and mechanism effects. (a) Plasma device consisting of a handheld with intensity adjust and different glass tubes to plug in. Formation of orange colored plasma when turned on. (b) Pictures taken with a thermal imaging camera after 0, 1, 2 and 4 minutes without any sign of heat development. (c) Ozone formation during time measured by an O_3_ detector demonstrating an increase of O_3_ concentration after 10 minutes already. The dashed line indicates the odor threshold concentration for ozone (20‒40 µg/m^3^; 0.01‒0.02 ppm).

In this study, *Signstek Portable High Frequency Machine* (Signstek, Full Rise LLC, Wilmington, DE/USA) carrying CE labeling (approved for use in humans in Europe) was used as a technical device consisting of a handheld with an intensity regulator on the bottom and an output for plugging in different glass tubes filled with helium below atmospheric pressure. Four different glass tubes are supplied: a comb tube for treating the scalp, a tongue tube for sensitive areas (such as dark circles beneath the eyes, nose, or lips), a mushroom tube for large areas (forehead, back), and a bent tube with a spherical tip for regular areas like cheeks (Fig. 1a).

In all experiments here, the bent tube was used at a working frequency of 50 Hz. The intensity was regulated at 50%.

### Technical measurements

Current strength was measured for three conditions (low, medium, and full power) using a standard ammeter (Multimeter ABB Metrawatt 2012). Applied voltage corresponding to the electric outlet (in Germany: 230 V).

### Evaluation of heat development

For further investigations on the precise mechanism of action of our plasma source, a thermal imaging camera (FLIR MS Technology C3, FLIR® Systems, Inc., Wilsonville, Oregon, USA) was utilized to detect potential heat development. Pictures were taken after 1, 2, and 4 minutes of HF treatment of an agar plate to resemble an experiment on bacteria.

### Ozone formation

Ozone (O_3_) concentration was measured using an O_3_ detector (Leory PM 2.5 O_3_ Ozondetektor TVOC, Thingnovation, Shenzhen, China). Our plasma source was switched on to 100% in a room with locked windows and doors of approximately 10.5 cubic meters. Measurement time points were 10, 20, 30, 40 and 50 minutes.

### Microbiology

#### Bacteriology

To study the effects of HF therapy on bacteria, swabs were taken from healthy skin in three different body areas (forehead, forearm, and genital area) from a healthy individual. In a second run, *Propionibacterium acnes* (ATCC® 6919™) was tested separately.

#### Mycology

To further evaluate the effects on fungi, clinical isolates of *Trichophyton* (T.) *mentagrophytes*, *T. violaceum*, *T. mentagrophytes*, *T. benhamiae*, *T. rubrum* and *Microsporum* (M.) *canis* determined by medical-laboratory assistants were treated as mentioned below.

### Cultivation of bacteria/fungi

Skin flora from three different body areas was isolated on blood agar plates and incubated for 24 h at 37 °C before HF treatment.

*P. acnes*, as an anaerobic bacterium, was cultivated in an anaerobic vial (BD BACTEC™ Lytic/10 Anaerobic/F Culture Vial) until an approximate cell density of 3.0 × 10^8^ CFU/mL referring to McFarland Standard nº 1 was reached, then following HF treatment on a blood agar plate. After treatment, plates were incubated for 7d at 37 °C in anaerobic conditions using BD GasPak™. Fungal species were cultivated on BD Dermatophyte Agar for 7d at 37 °C before treatment.

### In vitro model for high frequency testing in bacteria/fungi

After incubation, each investigated colony was resuspended in approximately 0.5 mL of sterile 0.9% saline solution. For skin flora, the authors chose five morphologically different appearing colonies for further evaluation. Thereafter, 1 µL of the microbial solution was applied centrally to the appropriate agar plates in pairs. Next, the HF device with an attached, wipe-disinfected bent tube was fixed on a stand at a distance of 5 mm above the plate. Of each pair, one plate was treated for 1 minute, and the other one served as a control to determine the relative difference. In the case of *P. acnes*, treatment periods varied between 1 and 8 minutes (1, 2, 4 and 8 minutes of HF treatment). Subsequently, the microbial solution of treated and untreated plates was spread using sterilized glass beads followed by an incubation period of 1d for skin flora and 7d for *P. acnes* and fungi at 37 °C. After incubation, on plates with more than approximately 1000 colonies, three 1 cm² areas on each agar plate were counted, and mean values were multiplied by the size of the agar plate (57 cm²) to obtain the absolute number of colonies per plate. Plates with 100 to 1000 colonies were divided into quarters and one counted quarter was multiplied by four, plates with less than 100 colonies were counted completely.

### 16S sequencing

Bacterial colonies of each plate pair were further characterized by performing a PCR (HotStarTaq Master Mix Kit, Qiagen) as described previously.^9^

Primers 27f (5’-AGA GTT TGA TCA TGG CTC A-3’) and 1492 r (5’-TAC GGT TAC CTT GTT ACG ACT T-3’) partially amplified the 16S rRNA gene using a thermocycler (Biometra Professional Basic, Analytik Jena GmbH, Jena, Germany).

The DNA products were visualized by 1.0% agarose gel electrophoresis to confirm a successful amplification. Sanger sequencing was performed by Eurofins Genomics (Ebersberg, Germany) using the 27f primer. Obtained sequences were aligned using the online tool BLASTn to identify the species (https://blast.ncbi.nlm.nih.gov/Blast.cgi; NCBI).^10^

### Statistical analysis

For visualization and statistical analyses (unpaired Student’s *t*-test and row test), GraphPad Prism (version 9.0.0, GraphPad Software Inc, San Diego, CA/USA) was used and data was visualized in logarithmically scaled graphs. All experiments were performed in triplicates. The following values are indicated by mean ± standard deviation. The significance level was set at p < 0.05 (*), p < 0.01 (**), and p < 0.001 were considered highly significant (***).

## Results

### Assessment of technical data and mechanism of action

To evaluate electrical current strength during treatment, a multimeter was used showing a current ranging from 0.28 (full power) to 0.32 ampere (low power) at a voltage of 230 V.

Heat development after 1, 2, and 4 minutes of HF treatment were not observed. Pictures taken with a thermal imaging camera did not show changes in temperature during the investigated period (Fig. 1b).

Ozone formation with a concentration of 0.015 ppm was observed after 10 minutes of a fully switched-on device. After 20 minutes, O_3_ concentrations almost doubled (c = 0.027 ppm). In the further course, concentration levels increased (c = 0.032 after 30 minutes, c = 0.040 after 40 minutes, c = 0.090 after 50 minutes) (Fig. 1c).

### Effects of high frequency therapy against skin flora

To assess the efficacy of HF therapy against common skin flora, blood agar plates with and without HF treatment were counted after the incubation period followed by a PCR to specify bacterial species. In broad terms, for almost every identified species a significant reduction in colony count was observed. More precisely, on the forehead, the authors detected *Micrococcus yunnanensis* twice, *Aerococcus viridans*, *Staphylococcus epidermidis* and *Staphylococcus capitis*. *M. yunnanensis* was reduced by 67.5% (31267.0 ± 81.7 vs. 1016.3 ± 57.1; p < 0.001) and on the second plate by 12.5% (6405.0 ± 18.5 vs. 5605.3 ± 19.4; p < 0.001), *A. viridans* by 26.1% (9339.0 ± 72.1 vs. 6906.3 ± 58.8; p < 0.001), *S. epidermidis* by 83.43% (3785.7 ± 68.7 vs. 627.3 ± 18.2; p < 0.001) and *S. capitis* by 41.9% (1488.0 ± 70.4 vs. 864.3 ± 30.3; p < 0.001).

Likewise for bacteria of the forearm: *B. cereus* was reduced by 75.9% (11396.3 ± 757.7 vs. 2750.7 ± 127.9; p < 0.001), *S. capitis* by 63.9% (2700.3 ± 104.0 vs. 973.7 ± 25.1; p < 0.001), *A. urinaeequi* by 95.1% (16201.0 ± 757.9 vs. 800.0 ± 35.0; p < 0.001), *M. yunnanensis* by 99.0% (7209.3 ± 344.8 vs. 75.0 ± 12.8; p < 0.001) and *M. luteus* by 3.1% (6280.0 ± 443.3 vs. 6087.7 ± 119.3; p = 0.5).

**Fig. 2:**
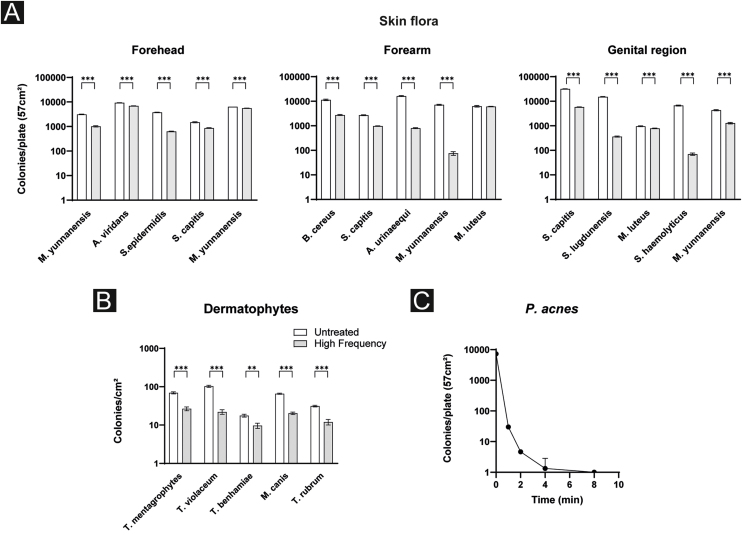
Antimicrobial effects of HF therapy. (a) HF treatment shows a significant decrease of bacterium and (b) fungus count in vitro. (c) CFU of *P. acnes* are significantly reduced after 1 minute, continuing the treatment leads to further reduction.

Regarding bacterial species of the genital region, *S. capitis* was reduced by 81.6% (31699.3 ± 763.7 vs. 5839.7 ± 111.3; p < 0.001), *S. lugdunensis* by 97.6% (15214.3 ± 405.1 vs. 363.3 ± 16.2; p < 0.001), *M. luteus* 17.9% (957.0 ± 37.6 vs. 785.3 ± 22.3; p = 0.002), *S. haemolyticus* 99.0% (6684.0 ± 273.0 vs. 70.0 ± 7.2; p < 0.001), *M. yunnanensis* 70.3% (4322.0 ± 252.3 vs. 1284.3 ± 86.4; p < 0.001) (Fig. 2a).

### Effects of high frequency therapy against dermatophytes

The next step of this study was to further evaluate the effects of HF treatment against a random selection of dermatophytes. The total number of all dermatophytes’ colonies was significantly reduced by 58.0% after HF treatment (55.8 ± 33.6 vs. 18.2 ± 6.5; p = 0.040). More precisely, in *T. mentagrophytes* colonies were reduced by 61.5% (69.3 ± 4.0 vs. 26.7 ± 3.1; p < 0.001), in *T. violaceum* by 78.6% (103.0 ± 6.6 vs. 22.0 ± 3.0; p < 0.001), in *T. benhamiae* by 45.2% (17.7 ± 1.5 vs. 9.7 ± 1.5; p = 0.003), in *M. canis* by 69.1% (65.7 ± 2.1 vs. 20.3 ± 1.5; p < 0.001) and in *T. rubrum* by 61.6% (31.3 ± 1.5 vs. 12.0 ± 2.0; p < 0.001) (Fig. 2b).

### Effects of high frequency therapy against *P. acnes*

To further address the question of whether HF treatment is beneficial against *P. acnes*, *P. acnes* was treated by HF over time (Fig. 2c). CFU on different blood agar plates was counted before and after 1, 2, 4, and 8 minutes of HF treatment. Significant results were reached after 1 minute of treatment (7288.0 ± 145.9 vs. 30.0 ± 3.6; p < 0.001). Continuing the treatment for 2 minutes, 4- and 8-minutes lead to a further decrease in the amount of CFU. When comparing the values after 2 and 4 minutes of treatment a further significant reduction of *P. acnes* CFU was observed (4.7 ± 0.6 vs. 1.3 ± 1.5; p = 0.020). Prolonged treatment over 8 minutes seemed not to have further benefits (1.3 ± 1.5 vs. 1 ± 0; p = 0.700) (Fig. 2c).

## Discussion

In this study, HF treatment demonstrated a microbicidal effect on skin flora and pathogens in vitro with a significant decrease in bacterial and fungal counts after a short treatment period.

While the authors show that HF devices do not work through heat development, we observed increasing O_3_ formation during the time of application suggesting its main antimicrobial effects are likely mediated by the development of ozone gas and therefore oxidative stress for the microbes. O_3_ is known to have an anti-inflammatory effect with antimicrobial properties.^11^, ^12^ In high concentrations (usually >200 µg/m^3^, >0.1 ppm) over prolonged periods of time, O_3_ might contribute to the development of lung diseases, such as COPD or emphysema,^13^, ^14^ but the amount of O_3_ in HF treatment is too small and time of application too short to cause serious health issues. The authors were able to show that skin flora from three different body areas including different aerobic bacteria was significantly reduced after 1 minute of HF treatment. In accordance with that, a previous study demonstrated a complete eradication of bacterial wound isolates in vitro.^8^ Furthermore, the present results are concordant with the findings of a study from 2015 showing that CAPP and VW can reduce a variety of bacteria, including Multi-Resistant Strains like oxacillin-resistant *Staphylococcus Aureus* (MRSA) and vancomycin-resistant *Enterococci* (ESBL) as well as fungi, like the yeast *Candida albicans*. The antimicrobial effects of VW did not relevantly differ from modern HF devices.^7^ The present study and previous studies clearly highlighted the antiseptic properties of HF therapies, thus suggesting that HF instead of antibiotics might be an alternative antimicrobial treatment option for easily accessible superficial infections, especially since multi-drug resistant bacterial strains are increasing. Moreover, the antimicrobial effect is also important for the reduction of *P. acnes* colonization as the authors were able to show a significant reduction after 1 minute of treatment with the HF device. Since *P. acnes* is a key player in acne vulgaris pathogenesis its reduction might display an important complemental treatment option.

## Conclusion

The results collected in this study, although in vitro, provide a mechanistic basis for HF as a complementary treatment option for patients with acne. It might also have a beneficial effect on patients with superficial infectious skin disorders. As the in vitro results are promising, further in vivo studies are needed to prove efficacy and tolerability in a real-life setting with long-term follow-ups to monitor possible side effects.

## Financial support

None.

## Authors’ contributions

Leonie Frommherz: Collected and analysed the data and drafted the manuscript.

Markus Reinholz: Collected and analysed the data and drafted the manuscript.

Anne Gürtler: Collected data, reviewed them and the manuscript.

Pia-Charlotte Stadler: Collected data, reviewed them and the manuscript.

Till Kaemmerer: Collected data, reviewed them and the manuscript.

Lars French: Collected data, reviewed them and the manuscript.

Benjamin M. Clanner-Engelshofen: Collected and analyzed the data and drafted the manuscript.

## Conflict of interest

None declared.
